# Nitrogen and Sulphur Fertilisation for Marketable Yields of Cabbage (*Brassica oleracea* L. var. *Capitata*), Leaf Nitrate and Glucosinolates and Nitrogen Losses Studied in a Field Experiment in Central Slovenia

**DOI:** 10.3390/plants10071304

**Published:** 2021-06-27

**Authors:** Nina Kacjan Maršić, Ksenija Sinkovič Može, Rok Mihelič, Marijan Nečemer, Metka Hudina, Jerneja Jakopič

**Affiliations:** 1 Biotechnical Faculty, University of Ljubljana, SI-1000 Ljubljana, Slovenia; s.ksenija@gmail.com (K.S.M.); metka.hudina@bf.uni-lj.si (M.H.); jerneja.jakopic@bf.uni-lj.si (J.J.); 2 Jožef Stefan Institute, Jamova 39, SI-1000 Ljubljana, Slovenia; marijan.necemer@ijs.si

**Keywords:** cabbage cultivation, nitrogen and sulphur fertilisation, nitrate content, glucosinolates profile, nitrogen surplus

## Abstract

A field trial of white cabbage (*Brassica oleracea* var. *Capitata* L.) was carried out under the humid temperate climate conditions in Central Slovenia to investigate the effects of calcium ammonium nitrate (0, 180 and 240 kg N ha^−1^) and gypsum (0 and 40 kg S ha^−1^) fertilisation on yield, yield quality (nitrate, glucosinolate levels and glucosinolate profile) and nitrogen use efficiency. The highest marketable yield, dry matter yield and nitrogen uptake were obtained at the highest nitrogen fertilisation rate when in combination with sulphur. For this treatment, the nitrogen surplus in the soil after harvesting was lower than for the same nitrogen fertilisation without sulphur application. For the combination N_240_S_40_, the sulphur addition significantly increased nitrogen use efficiency, which resulted in reduced nitrate content in the cabbage heads. The chemical forms of glucosinolates showed that 80–85% were aliphatic glucosinolates with the remainder as the indole group. For the aliphatic glucosinolates, significant interactions between nitrogen and sulphur fertilisations were reflected in increased levels of progoitrin and glucoiberin when sulphur was applied at the lower nitrogen fertilisation rates. For the indole group, the levels of glucobrassicin and the indole group itself decreased at higher nitrogen fertilisation rates, independent of sulphur fertilisation.

## 1. Introduction

As an economically important members of the Brassicaceae family, white cabbage (*Brassica oleracea* var. *Capitata* L.) is consumed worldwide and is considered a good source of bioactive phytochemicals [1]. The most striking feature of such Brassica crops is their high levels of glucosinolates [2], which have chemoprotective and anticancer effects after myrosinase hydrolysis [3]. These properties of glucosinolate metabolites have motivated recent efforts to better understand the factors that influence their production in plants [4,5,6,7].

The classification of glucosinolates depends on the amino acid precursor in the side chain, and these molecules contain at least two sulphur atoms, which is the main reason for the high sulphur demand of Brassica crops [8]. Glucosinolates are known to be related to plant defence mechanisms, as they are induced after injury by herbivores or pathogens, and changes in various environmental factors, such as temperature, radiation and salt and nutrient content [1,9,10,11]. In addition to these the environmental effects, fertilisation with nitrogen and/or sulphur has been shown to have significant effects on the glucosinolate levels in plant tissues [9,10].

The application of nitrogen fertilisers is essential for the formation of primary and secondary metabolites in plants, and together with carbon and sulphur, nitrogen is an important source of protein synthesis, growth and phytochemical biosynthesis. As there is also nitrogen in glucosinolates, it can be assumed that their levels increase when plant growth is limited by carbon availability, with the excess nitrogen being channelled into these secondary metabolites [12].

Over the last three decades, increasing sulphur deficiency in soils has been reported for many European countries. This is mainly due to the reduction in sulphur dioxide emissions from industrial sources, the increasing use of low sulphur fertilisers and the increase in crop yields due to the fact of technological advances [13]. Thus, sulphur has become one of the most limited nutrients in agricultural production, especially for Brassica crops. In particular, in higher plants, inorganic sulphur is assimilated into cysteine, which is subsequently converted into methionine [14]. The activities of the enzymes involved in these reduction steps are regulated by the nitrogen content in the plant [15,16].

To satisfy the increasing health and environmental awareness of consumers, the demand for vegetables with high levels of health-promoting phytochemicals produced by sustainable production systems need to be fulfilled [17]. However, it is often very difficult to satisfy environmental sustainability in vegetable production, as cultivation systems are highly vulnerable to nitrogen loss (e.g., nitrate leaching, denitrification). This is largely due to the conditions of high nitrogen input, frequent cultivation, relatively short periods of plant growth and low nutrient use efficiency [18]. Various efforts to reduce fertilisation rates without compromising yield have been evaluated in white cabbage, based on improved nitrogen and water management practices [19,20,21,22], development of various breeding programmes that focus on nitrogen-efficient genotypes [23] and improved models to predict the timing of nitrogen uptake [24].

The effects on the nutritional quality of Brassicaceae plants through nitrogen and/or sulphur supplies and nutrient uptake, as well as their efficient use, have previously been studied separately [11,20,21,25,26]. Thus, there are few data available on the combined effects of fertilisation and nutrient use efficiency (NUE) on health-promoting compounds and the nutritional value of plants in a sustainable environment [11,26]. Furthermore, the relationships between glucosinolates in plants and nutrient availability, especially N and S, have been studied mainly on oilseed Brassica crops [27,28,29,30,31,32] or broccoli, turnip and mustard [11,33,34,35], while for cabbage such data are very scarce in the literature [9].

The objective of this study was to investigate the effects of nitrogen and sulphur fertilisation on cabbage yield and quality (as nitrate content and glucosinolate levels), nitrogen uptake and NUE and the potential for nitrogen loss on sandy loam soils under the humid temperate climatic conditions of Central Slovenia. A field trial was thus conducted to evaluate the fertilisation regime that provides high qualitative and economic yields and low potential for nitrogen loss (i.e., low postharvest soil mineral nitrogen surplus).

## 2. Results

### 2.1. White Cabbage Yield and Nitrogen Uptake 

For the non-fertilised control, the marketable yield of these cabbages was 30.3 ± 1.6 t ha^−1^. Fertilisation with 180 kg N ha^−1^ and 40 kg S ha^−1^ (N_180_S_40_) more than doubled this yield to 67.4 ± 2.3 t ha^−1^. The yield was then further increased by increasing the nitrogen fertilisation from 180 to 240 kg ha^−1^ to 75.8 2.6 t ha^−1^, although only when sulphur was also applied with the nitrogen (N_240_S_40_) (Table 1). The mean head weight of the cabbages was also higher with this treatment (N_240_S_40_) than without sulphur addition (N_240_S_0_).

No significant effects of nitrogen and/or sulphur application was observed on the proportion of marketable yield, which ranged from 70% to 78% (Table 1). Dry matter yield was higher in treatments with high nitrogen accompanied by sulphur (N_240_S_40_; 8050 ± 871 kg ha^−1^), compared to high nitrogen fertilisation without sulphur (N_240_S_0_; 7113 ± 274 kg ha^−1^) (Table 2).

### 2.2. Nitrogen, Sulphur and Nitrate Contents in the Tissue of Cabbage Heads

Total nitrogen content in the cabbage head tissue and in the wrapped leaves was only affected by nitrogen application, without any significant effects of addition of sulphur (Table 3). The nitrogen content increased in the heads of the control treatment from 15.2 ± 0.6 g·kg^−1^ dry matter to 27.8 ± 0.4 g·kg^−1^ dry matter in the heads fertilised with high nitrogen (N_240_S_40_ and N_240_S_0_). For the wrapped leaves, the nitrogen contents with the N_240_S_40_ treatment were 40% higher and 65% higher with the control plants compared to the cabbage heads under the corresponding treatments.

The sulphur content was significantly higher in cabbage heads when sulphur was applied with the nitrogen fertilisation, which resulted in a 13% increase in sulphur content for the low nitrogen (N_180_S_40_) and a 30% increase for the high nitrogen (N_240_S_40_) (Table 3). Nitrogen fertilisation also significantly increased sulphur content in the head tissue, although only when it was supplied together with sulphur. This was reflected in a 9% increase in the cabbage head sulphur content when nitrogen was increased from 180 to 240 kg N ha^−1^.

Nitrate content in heads and wrapped leaves was also affected by nitrogen and sulphur fertilisation (Table 4). The highest nitrate content was in the heads and wrapped leaves for the high nitrogen without sulphur (N_240_S_0_; 1816 ± 282, 3127 ± 395 mg NO_3_^–^ kg^−1^ FW, respectively). The addition of sulphur at this high nitrogen significantly reduced the nitrate content in both of these cabbage parts, with a nitrate reduction in the head tissue by 25%, and in the wrapped leaves by 28%.

### 2.3. Balance of Nitrogen Input and Output

The calculated nitrogen budget for these fertilised plots indicated that the nitrogen input exceeded the nitrogen uptake by the plants, which resulted in excess nitrogen in the soil (Table 5). The extent of the nitrogen surplus varied according to the fertilisation. The treatments with high nitrogen application without sulphur (N_240_S_0_) showed the highest nitrogen surplus (almost 53 kg N ha^−1^), while the plots where high nitrogen application was accompanied by sulphur (N_240_S_40_) showed reduced nitrogen surplus (19 kg N ha^−1^). The addition of sulphur to the standard nitrogen application rate also resulted in a reduction in the nitrogen surplus, from 15.0 (N_180_S_0_) to only 2.2 kg N ha^−1^ (N_180_S_40_).

### 2.4. Glucosinolates Levels in Cabbage Heads

Nine individual glucosinolates were quantitatively determined for the cabbage heads: the aliphatic glucosinolates sinigrin, gluconapin, glucoiberin, progoitrin and glucoibervirin; the indole glucosinolates 4-hydroxyglucobrassicin, glucobrassicin and neoglucobrassicin; the aromatic glucosinolate gluconasturtiin (Table 6). Total glucosinolates was calculated as the sum of these individual glucosinolates.

Although no significant differences were seen for the total glucosinolates, the standard nitrogen rate with sulphur showed the highest levels (N_180_S_40_; 30.0 ± 3.1 µmol g^−1^ DW), with similarly low levels seen across a number of the treatments (N_180_S_0_; N_240_S_0_; N_240_S_40_; ~24 µmol g^−1^ DW) (Table 6). Significant effects of nitrogen and sulphur were seen in the levels of two of the individual aliphatic glucosinolates (i.e., glucoiberin and progoitrin) and in one of the indole glucosinolates (i.e., glucobrassicin). Fertilisation at standard nitrogen without sulphur and at high nitrogen irrespective of sulphur showed significant reductions in progoitrin compared to the control. The glucoiberin levels were lowest for both of the nitrogen fertilisation treatments without sulphur. The glucobrassicin levels decreased compared to the control treatment when standard nitrogen was applied without sulphur, while fertilisation with high nitrogen reduced glucobrassicin levels regardless of sulphur. Glucobrassicin levels also changed according to sulphur, but only at the standard nitrogen rate, with sulphur fertilisation increasing glucobrassicin levels by up to 44%.

## 3. Discussion

### 3.1. Effects of Nitrogen and Sulphur Fertilisation on Yields

The cabbage yields in this study at least doubled with fertilisation compared to the unfertilised control. This indicates that fertilisation contributed more to cabbage yield than for other crops, where similar fertiliser application has been shown to increase yields by approximately 60%, mainly for cereals and oilseeds in China and Europe [36,37]. Increasing the nitrogen from 180 to 240 kg N ha^−1^ also resulted in increased cabbage yield, but only when sulphur was applied together with the nitrogen, which occurred in other studies with different Brassica crops [10,38,39].

As both of these nutrients are involved in the biosynthesis of proteins and many other important biomolecules, this balanced application of nitrogen and sulphur can increase their efficiency of use in crops [40]. The relationships between the roles of nitrogen and sulphur in plants can be observed in the repression of the activity of the enzyme nitrogen reductase [41], which catalyses the rate-limiting step in the nitrate assimilation pathway. This is also apparent for the regulation of ATP–sulphur lyase when nitrogen-limitation or sulphur-deficient conditions occur in plants [40,42,43]. Moreover, the synthesis of cysteine as a result of the incorporation of the sulphide moiety into O-acetylserine appears to be the meeting point between nitrogen and sulphur metabolism. Naturally occurring thiol compounds (e.g., cysteine, glutathione) have also been shown to affect nitrate reductase activity in Brassica plants [44].

### 3.2. Effects of Nitrogen and Sulphur Fertilisation on the Nitrogen Balance in Soil and Plants

Calculation of the nitrogen budget under the experimental conditions in the present study shows a potential risk for nitrogen loss following treatments with high nitrogen (i.e., 240 kg N ha^−1^) compared to standard nitrogen (i.e., 180 kg N ha^−1^) [20,21]. However, nitrogen surpluses were reduced when the gypsum sulphur was added with the nitrogen and even more so at the high nitrogen application.

However, the calculated nitrogen surpluses of all of these treatments were relatively small. Under the average meteorological conditions in such Slovenian agricultural areas, there would only be a potential threat of nitrate pollution of the ground water for a nitrogen excess of >45 kg N ha^−1^ [45]. Thus, even the highest nitrogen surplus in the present study of 52.7 kg ha^−1^ at N_240_S_0_ remained relatively low. The mean nitrogen surplus overall for agriculture in Slovenia has been reduced over the last 15 years, from the previous 98 to 42 kg N ha^−1^ per year [46,47]. However, these data were based on all land that was used for agriculture and, thus, also poorly or non-fertilised marginal land; these data will thus not specifically represent the nitrogen surplus of the intensively cultivated land in the present study.

The NUE across all of the treatments in the present study was high, even for the lowest NUE seen for N_240_S_0_, at 78%. Indeed, this can be considered optimal, as proposed by the model of the panel of European experts on nitrogen fertilisation. They indicated that in common agricultural practice, NUE > 80% can lead to soil nitrogen mining and to long-term worsening of soil fertility [36]. Here, high NUE was obtained for the combination of nitrogen with gypsum (i.e., sulphur), as 98% for N_180_S_40_, and 92% for N_240_S_40_. Considering the data obtained here under these environmental conditions and soil types, even the high nitrogen combined with sulphur (N_240_S_40_) left only 19 kg N ha^−1^ surplus, indicating that this economically valuable yield can be obtained without great environmental risk. This is particularly important, because most of the highly fertile arable land in Slovenia is located on plains in shallow groundwater recharge zones that are the main source of drinking water [20,48]. The relationships between the supplies of nitrogen and sulphur have been noted in many studies, as the uptake of these nutrients is strongly linked due to the fact of their central role in plants as mentioned earlier [2,10,43]. Therefore, a shortage of sulphur in plants is likely to result in less use of the available soil nitrogen and, thus, to an increased risk of nitrate leaching [44].

As well as fertilisation and other agricultural practices, the data obtained here will have been dependent also on the weather conditions, which were particularly favourable during this study, thus promoting further the high yield with good responses to fertilisation. However, for less favourable years, which include drought periods or when farmers did not fertilise with sulphur along with nitrogen, we would recommend that nitrogen fertilisation of early cabbage varieties remains closer to the standard nitrogen rate of 180 kg N ha^−1^ to reduce any adverse environmental impact. Similarly, for the same reason, it has been suggested that for late-growing cabbage varieties [49,50], the recommended fertilisation rate of 350 kg N ha^−1^ for cabbage can be reduced without reducing the crop yield.

### 3.3. Effects of Sulphur and Nitrogen Fertilisation on Nitrate Content in Cabbage

The beneficial effects of sulphur fertilisation were reflected not only in the more efficient uptake of nitrogen in the soil but also in the more efficient nitrogen use in the plants. This was reflected in the lower nitrate content in the cabbage heads for the treatments with nitrogen combined with sulphur, compared to nitrogen without sulphur. The differences in the nitrate contents were significant in the cabbage heads fertilised with the high nitrogen rates, which thus confirmed data from other studies [11,43,51]. Indeed, sulphur application promoted the incorporation of nitrogen into organic compounds, and consequently reduced the nitrate content in the leaf. For all of these nitrogen and sulphur treatments, the nitrate content in the cabbage heads were lower than those reported for other leafy vegetables [10,52,53].

### 3.4. Effects of Nitrogen and Sulphur Fertilisation on Levels of Eight Glucosinolates in Cabbage Heads

In this study, the indole glucosinolates accounted for approximately 13–19% of the total glucosinolates, while the aliphatic glucosinolates accounted for approximately 80–87%. These are not consistent with previous studies with cabbage plants, in which indole glucosinolates have accounted for more like 80–85% [9,17,32]. These contrasting data might be due to the different cultivars used and the different environmental conditions. Indeed, higher amounts of alkyl glucosinolates are synthesised at lower temperatures in combination with increased radiation, and high amounts of the indole glucosinolate glucobrassicin have been reported for higher temperatures (>18 °C) and lower radiation [11]. The cabbage plants in the present study were grown from April to July, when the mean daily temperatures ranged from 12 to 19 °C, and the mean daily radiation increased a little (from 15 to 42 mol m^–2^ day^−1^), which suggests that the growing conditions had major effects on the structures of the glucosinolates produced in the head tissue of these cabbage plants.

These results are important for growers and researches in most leading cabbage producing countries (South Alpine region of Europe, as well as parts of China, India, Russia and Poland) where cabbage production mainly takes place in regions with temperate, humid climatic conditions, [54,55,56,57], as they characterised the climate in this study.

The predominance of sinigrin and glucobrassicin in cabbage plants has been reported previously [9,58], while the amount of the aromatic glucosinolate gluconasturtiin, which was detected in the present study, has not yet been demonstrated in cabbage plants. Considering the important anticarcinogenic activity of the degradation products of sinigrin, glucobrassicin and gluconastrurtiin [2,59,60], consumption of cabbages cultivated in early summer is recommended for a healthy diet. Fertilisation with nitrogen and sulphur showed differential effects on the individual aliphatic glucosinolates and indole glucosinolates in the present study. The addition of sulphur significantly affected two of the aliphatic glucosinolates, while the addition of nitrogen resulted in significantly decreased levels of glucobrassicin and the total indole group glucosinolates. Furthermore, at the standard nitrogen fertilisation rate, the glucosinolates responses were dependent on the supply of sulphur. Effects on increasing glucosinolates levels from increasing sulphur fertilisation have been reported previously for numerous crucifers grown in low sulphur soils [9,11,61], although these were only partially consistent with the present study. The lack of significant changes in sinigrin levels and the total aliphatic group in the present study might be because the sulphur content in the soil was high enough already to saturate the plant requirements for glucosinolates biosynthesis, which would thus mask any effects of the additional sulphur on glucosinolates synthesis. Indeed, the wet deposition of sulphur in Slovenia is the highest among European countries, at 9.8 kg S ha^−1^ year^−1^ [62]. Nevertheless, similar results for fertiliser’s effect on quality parameters in cabbage plants could be expected for studies conducted in Europe countries and China, where similar pedoclimatic conditions are regulated by temperate continental climate and sufficient rainfall, both of which best suit the growth requirements of cabbage plants [13,62,63].

The data for the indole glucosinolates levels here indicated that nitrogen and sulphur have important roles in the regulation of glucosinolates synthesis in cabbage heads. The indole glucosinolates levels were high when the nitrogen and sulphur were not at adequate levels (i.e., control, unfertilised plants), and these decreased with increasing nitrogen fertilisation. When the nitrogen was added at above the standard nitrogen rate (S_240_), and regardless of the sulphur, the indole glucosinolates levels decreased, which is consistent with previous studies [9,29,64]. This might be the consequence of sulphur deficiency in the synthesis of glucosinolates in plants that was caused by the high nitrogen fertilisation. Indeed, for broccoli and oilseed rape, it has been shown that for glucosinolates synthesis, for every 10 (or even fewer) parts nitrogen, there is one part sulphur [65]. Schonhof et al. reported [11] that a higher nitrogen:sulphur ratio resulted in lower glucosinolates production in broccoli heads, which is partially consistent with the present study. Here, the increase in nitrogen fertilisation resulted in an increase in the nitrogen:sulphur ratio (N_0_S_0_, 5.2 (control plants); N_180_S_0_, 6.5; N_240_S_0_, 7.6) and a decrease in the indole levels (4.8–3.7 µmol g^−1^ DW).

Indole glucosinolates are derived from the sulphur-free amino acid tryptophan, and their levels do not normally change due to the fact of sulphur fertilisation, as is seen for the aliphatic glucosinolates, which are derived from the sulphur-containing amino acid methionine [14,66]. However, sulphur fertilisation in the present study increased the indole glucosinolates in the cabbage plants grown at the standard nitrogen fertilisation rates (180 kg N ha^−1^). These data are in agreement with previously reported suggestions that an increased sulphur supply increases glucosinolates levels in Brassica plants, as these plants assimilate inorganic sulphate into cysteine, which is subsequently converted to methionine, where this reduction step is regulated by nitrogen [5,66]. Moreover, the synthesis of the indole glucosinolates from tryptophan is dependent on the thiohydroximate sulphur donor (i.e., cysteine or methionine) as a precursor of indole glucosinolates [14].

In further studies with cabbage plants, a cultivation site with low soil sulphur should be selected and a wider range of nitrogen and sulphur fertilisation rates should be used to obtain the clearer effects of nitrogen and sulphur fertilisation on glucosinolates accumulation in white cabbage and to determine the rate at which the yield increases to the point where fertilisation does not result in significant negative environmental effects.

## 4. Conclusions

Increased nitrogen fertiliser up to 240 kg N ha^−1^ combined with a sulphur supply resulted in higher cabbage yields and nitrogen uptake. Among the fertiliser rates tested in this study, and under the given experimental conditions, the combination of 240 kg N ha^−1^ with 40 kg S ha^−1^ reduced the nitrogen surplus after harvest and was the most suitable for cabbage cultivation from both the economical (yield) and ecological (leaching potential) points of view. For the plants grown at the high nitrogen rate (240 kg N ha^−1^), sulphur addition increased NUE, which was reflected in lower nitrate content in the cabbage heads. The structures of the glucosinolates in the cabbage heads showed that the predominant glucosinolates belong to the aliphatic group, where their synthesis is stimulated by lower temperatures and increased light conditions, which is consistent with the growing conditions in the present study, and which indicates an important role for the growing conditions in terms of glucosinolates synthesis. The nitrogen and sulphur fertilisation affected the levels of some of the glucosinolates. The interactions of nitrogen and sulphur fertilisation were significant for glucoiberin and progoitrin levels for the aliphatic glucosinolates and for the glucobrassicin levels from the indole glucosinolates. This was reflected in increased levels with the low nitrogen rate when sulphur was also applied. Nitrogen fertilisation resulted in decreased indole glucosinolates levels, probably due to the sulphur deficiency for glucosinolates synthesis as a result of the increased nitrogen fertilisation.

## 5. Materials and Methods

### 5.1. The Field Experiment: Site Description, Soil Properties and Weather

The experiment was conducted in an experimental field at Dolnje Brezovo (45°59′38″ N, 15°22′21″ E; 170 m a.s.l.), approximately 67 km east of Ljubljana, the capital of Slovenia. The field is part of 40 ha of agricultural land where intensive vegetable production has been ongoing for nearly 30 years. Soil samples for determination of texture classification and basic nutrient analysis were taken before and after the experiment, and the physical and chemical properties of the soil are given in Table 7. The soil of the experimental site was classified as gleyic fluvisol and endogleyic fluvisol, according to the World References Base for Soil Resources (2015). The laboratory analyses included soil texture, organic matter, pH, plant-available phosphorus (P_2_O_5_) and potassium (K_2_O) (ammonium lactate method) [67]. Determination of total sulphur in soil was carried out by energy-dispersive X-ray fluorescence (EDXRF) spectrometry [68].

**Figure 1:**
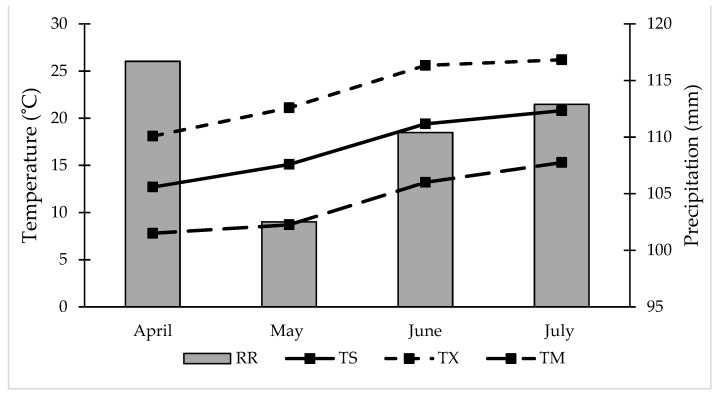
Monthly meteorological data from April to July 2014 for the Novo Mesto meteorological station. Data were obtained from the Environmental Agency of the Republic of Slovenia (ARSO, 2014). TS, mean monthly air temperature; TX, mean daily maximum temperature; TM, mean daily minimum temperature; RR, precipitation.

The mean annual precipitation in the study area for the 1981–2010 reference period was 1179 mm, and the average annual air temperature was 8.9 °C (ARSO, 2014), measured at the nearest representative meteorological station of Novo Mesto (45°48′6.5″ N, 15°10′38.3″ E; 220 m a.s.l.). Data for precipitation and air temperatures for the growing season are shown in Figure 1. During the growing period, the mean daily temperature was above the 30 year average by 1.6 °C in April and by 0.8–1.6 °C in May, June and July. Precipitation exceeded the 30 year average in total by 28 mm, with the greatest excess in April (28 mm above the average). In May and July, precipitation was 5 mm and 14 mm above the average, respectively, while in June it was 20 mm below the average.

### 5.2. Design and Management of the Field Experiment

The experiment was laid out in a randomised complete block design with four replications. Each block (replicate) consisted of five plots (fertilisation treatments). Each plot was 1.9 × 3 m², with 20 plants per plot. The distance between rows was 0.75 m, and between plants within rows, 0.38 m. Cabbage (*Brassica oleracea* var. *Capitata* L.) cv. ‘Nozomi F1’ (Sakata, Japan) transplants were purchased from the Dutch Transplant Organisation, and 35 day old transplants were transplanted into the field on 15 April 2014.

The five fertiliser treatments used were the unfertilised control plot (N_0_S_0_); 180 kg N ha^−1^ without (N_180_S_0_) and with 40 kg S ha^−1^ (N_180_S_40_); 240 kg N ha^−1^ without (N_240_S_0_) and with 40 kg S ha^−1^ (N_240_S_40_). Calcium ammonium nitrate (27% nitrogen) was used as the nitrogen source, and the gypsum product ‘Calcin S’ (20% calcium, 16% sulphur; Cinkarna Celje, Slovenia) was used as the source of sulphur. Phosphorus (as superphosphate, 14% P_2_O_5_) was added at the equivalent of 80 kg P_2_O_5_ ha^−1^, potassium (as KCl, 60% K_2_O) at the equivalent of 300 kg K_2_O ha^−1^. The nitrogen, phosphorus, potassium and sulphur fertilisers were manually distributed over the soil surface 1 day before transplanting. These amounts of the added fertilisers were based on the recommendations for the integrated production of vegetables [69].

The irrigation was managed according to local agricultural practices, i.e., 20 mm of water using tank sprinklers on the day before and the day after transplanting (DAT). During the experimental period, the irrigation regime was generally adjusted to the actual weather conditions in the field to avoid water stress.

### 5.3. Sampling and Sample Preparation

Cabbage heads were harvested 68 DAT, at the maturity stage as defined by the density of heads (i.e., heads were compact and hard when the top of the head is pinched by a finger, and the wrapped leaves that were tight against the heads curled up a little). Samples (six plants per repetition) were randomly taken from the centre of the experimental plot. The weight of each cabbage head with wrapped leaves and without wrapped leaves (i.e., marketable weight) was measured, and the total yield was calculated using the fresh head weight, with multiplication by the number of plants per square metre. The yield was expressed in tons per hectare, and 20% of the total yield was removed to account for the tractor wheel paths, where plants would not have been planted under normal field production conditions.

### 5.4. Nitrogen, Sulphur and Dry Matter Content of Plants

Total nitrogen and dry matter content of the plants were determined by harvesting six plants at maturity from each repetition of each treatment. The plants were divided into the subsamples of the edible part (i.e., heads) and non-edible part (i.e., wrapped leaves). The cabbage fractions were cut and mixed. Subsamples of about 500 g were dried at 60 °C for determination of dry matter content and subsequently analysed for total nitrogen, which was determined after incineration at 900 °C using a thermal conductivity detector (Elementar VarioMAX CN analyser; Germany) (ISO 13878). The measurement uncertainty was 9%.

The nitrate content in the fresh samples were determined in water extracts using a UV/Vis spectrometer (Lambda 2; Perkin-Elmer) with a flow injection analysis system (Neumann and Bassler, 1976). The measurement uncertainty was 17%.

Dry matter yield (kg ha^−1^) and nitrogen uptake (kg N ha^−1^) were calculated according to Equations (1) and (2), respectively.

(1) $$\mathrm{Dry}\mathrm{matter}\mathrm{yield}\;(\mathrm{kg}{\mathrm{ha}}^{-1})=\mathrm{FW}\;\left(\mathrm{kg}\right)\times \frac{10000}{\mathrm{Area}\mathrm{harvested}\;(m^{2})}\times \frac{\mathrm{SDW}}{\mathrm{SFW}}$$

(2) $$N_{\mathrm{uptake}}\;(\mathrm{kg}\;{\mathrm{ha}}^{-1})=\mathrm{Dry}\mathrm{matter}\mathrm{yield}\;(\mathrm{kg}{\mathrm{ha}}^{-1})\times \frac{\%\;N\;\mathrm{in}\;\mathrm{plant}\;\mathrm{tissue}}{100}\;$$

where FW (kg) is the fresh weight of the sample per area harvested, and SDW (kg) and SFW (kg) are the dry and fresh weights of the subsamples, respectively. The harvested area is in m^2^, and the value of 10,000 has the units of m^2^ ha^−1^. Total nitrogen uptake (kg ha^−1^) was calculated by multiplying the dry matter yield of the plant parts (i.e., head and wrapped leaves) by the corresponding nitrogen content in the plant parts.

Determination of total sulphur was carried out by non-destructive energy-dispersive X-ray fluorescence (EDXRF) spectrometry. The pellets were prepared with 0.5–1.0 g of the powdered samples using a pellet die and a hydraulic press. For excitation, a disc radioisotope excitation source of 55Fe was used (25 mCi; Eckert & Ziegler). The emitted fluorescent radiation was measured using EDXRF with a detector (XR-100SDD; Amptek), a digital pulse processor (PX5; Amptek) and a PC-based multichannel analyser software package (DPPMCA; Amptek). The spectrometer was equipped with a vacuum chamber. The energy resolution of the spectrometer was 125 eV at 5.9 keV. The complex X-ray spectra were analysed using the AXIL Spectral Analysis Programme [68,70]. This required the statistical uncertainties of the measured intensities and the uncertainties of the mathematical fitting procedure. For these purposes, quantification was performed using the Quantitative Analysis of Environmental Samples software that was developed in our laboratory [68,70]. The estimated uncertainties in the analysis were between 5% and 10%. The relatively high total estimated uncertainty was mainly due to the matrix correction and the geometry of the calibration procedures that included errors in the tabulated fundamental parameters. It might also have arisen from the effects of the spectrum acquisition and analysis. The accuracy of the data was checked using the National Institute of Standards and Technology reference materials: 1547 (peach leaves) and 1573a (tomato leaves).

### 5.5. Apparent N Recovery and Nitrogen Use Efficiency

The cumulative apparent nitrogen recovery (ANR) was calculated as the difference between the cumulative N removed (CNR) by the crop in each treatment less the control treatment, divided by the total cumulative N added (CNA) for the treatment, expressed as a percentage. This parameter provides an estimate of the efficiency of the applied fertiliser.

The NUE was defined in this study as the degree that the nitrogen input contributes to the nitrogen contained in the products. This was calculated as the total nitrogen contained in the products, divided by the total nitrogen input, including the fertiliser, as in Equation (3) [37]:

(3) $$NUE\;\left(\%\right)=\frac{\mathrm{Harvested}\;N}{\mathrm{Total}\;N\;\mathrm{input}\;\mathrm{to}\;\mathrm{cropland}\;}$$

### 5.6. Glucosinolate Analysis in the Plants

Extraction and desulphonisation of the glucosinolates was carried out according to the standardised method (International Standards Organisation, 1992). Briefly, the freeze-dried plant material was ground to a fine powder in liquid nitrogen. The powder (0.2 g) was heated to 70 °C for 3 min to inactivate endogenous myrosinase to thus prevent the degradation of the glucosinolates. This was added to 2 mL boiled 70% MeOH, and left for 10 min at 70 °C. The sample was then centrifuged at 10,000× *g* for 5 min (5810 R centrifuge; Eppendorf, Hamburg, Germany). The supernatant was collected in a centrifuge tube, and the sample was re-extracted with 2 mL of 70% boiled MeOH. After centrifugation, the second supernatant was added to the first in the centrifuge tube, and pure water was added to 5 mL. The supernatant was applied to a DEAE Sephadex A-25 column (500 mL), which was washed twice with 1 mL sodium acetate buffer (0.02 M at pH 4.0). Desulphation was carried out by the addition to the column of 75 µL diluted purified sulphatase solution, with the columns left overnight at ambient temperature. The columns were then eluted twice with 750 µL pure water, and the eluate was collected in a vial.

The individual glucosinolates were identified and quantified using high-performance liquid chromatography (Accela HPLC system; Thermo Scientific, San Jose, CA, USA) according to ISO 9167-1:1992 (International Standards Organisation, 1992). This system included a photodiode array detector and was controlled through the chromatography workstation software (CromQuest 4.0). The column (Gemini C18; 150 mm × 4.6 mm 3 μm; Phenomenex, Torrance, CA, USA) was operated at 30 °C. Mobile phases A (100% water) and B (20% acetonitrile in water; *v*/*v*) were applied from 100% A at 0 min to 100% B at 20 min and then isocratic for 5 min, before returning to the initial conditions. The flow rate was 1 mL min^−1^, the injection volume was 20 µL and the wavelength for determination was 229 nm.

The eluent from the HPLC was interfaced with a triple-quadrupole mass spectrometer (TSQ Quantum Access MAX; Thermo Scientific, San Jose, CA, USA) via a heated electrospray ionisation probe operated in positive ion mode at an operating temperature of 350 °C and a vaporiser temperature of 50 °C. High-purity nitrogen was used as the sheath (60 units) and auxiliary (15 units) gas. Positive ion tandem mass spectrometry was used to detect desulphoglucosinolates with selected reaction monitoring. The scan event cycle used a full scan mass spectrum with a range of m/z 200–650 and the corresponding data-dependent tandem mass spectrometry events.

The levels of the individual desulphoglucosinonates were calculated according to the response factors, and the levels are expressed as μmol equivalents of desulphosinigrin g^−1^ dry weight (DW).

### 5.7. Statistical Analysis

Two-way analysis of variance (ANOVA) was used for the effects of the nitrogen and sulphur fertilisation and their interactions on the parameters tested (i.e., cabbage yield, dry matter yield, nitrogen and sulphur contents in the plants, nitrogen uptake by the plants and nitrogen and nitrate distribution between the marketable parts of the plants and wrapped leaves), using the Statgraphics Centurion programme (Manugistics Inc., Rockville, MD, USA). Differences between treatments were estimated using Duncan’s multiple comparison tests at a significance level of *p* = 0.05.

## Figures and Tables

**Table 1:** Effects of nitrogen and sulphur fertilisation on the marketable yield parameters of white cabbage and the nitrogen and sulphur interactions.

Fertilisation (kg ha^−1^)		Mean Head	Marketable Yield	
Nitrogen	Sulphur	Weight (g)	(t ha^−1^)	(%)
0	0	860 ± 50 ^c^	30.3 ± 1.6 ^c^	70.3 ± 1.3
180	0	1790 ± 55 ^b^	62.7 ± 1.8 ^b^	76.2 ± 0.8
	40	1920 ± 60 ^b^	67.4 ± 2.3 ^b^	75.1 ± 0.9
240	0	1900 ± 70 ^b^	66.8 ± 2.4 ^b^	76.1 ± 0.6
	40	2160 ± 75 ^a^	75.8 ± 2.6 ^a^	78.1 ± 0.7
**Significance**				
Nitrogen		**	**	ns
Sulphur		ns	ns	ns
Nitrogen × Sulphur		**		ns

Data are means ± standard error (*n* = 4). Different superscript letters indicate significant differences between fertilisation treatments (*p* < 0.01; Duncan’s test). ** *p* < 0.01; ns, not significant (ANOVA), for interactions with and between nitrogen and sulphur fertilisations.

**Table 2:** Effects of nitrogen and sulphur fertilisation on dry matter yield and nitrogen measures for cabbage heads and wrapped leaves and the nitrogen and sulphur interactions.

Treatments		Dry Matter Yield		Nitrogen Uptake		Apparent Nitrogen Recovery		
(kg ha^−1^)		(kg ha^−1^)		(kg ha^−1^)		(%)		
Nitrogen	Sulphur	Heads	Wrapped Leaves	Heads	Wrapped Leaves	Heads	Wrapped Leaves	Together
0	0	3402 ± 167 ^c^	2500 ± 95 ^c^	52 ± 4 ^d^	63 ± 3 ^c^			
180	0	6750 ± 205 ^b^	3140 ± 174 ^b^	169 ± 7 ^c^	110 ± 4 ^b^	65	26	91
	40	7133 ± 262 ^b^	3465 ±110 ^a^	181 ± 8 ^bc^	128 ± 8 ^a^	72	36	108
240	0	7113 ± 274 ^b^	3274 ± 130 ^ab^	192 ± 7 ^b^	130 ± 5 ^a^	58	28	86
	40	8050 ± 271 ^a^	3330 ±128 ^ab^	225 ± 7 ^a^	129 ± 6 ^a^	72	28	100
**Significance**								
Nitrogen		***	***	**	**			
Sulphur		ns	ns	*	*			
Nitrogen × Sulphur		**	**	**	**			

Data are the means ± standard error (*n* = 4). Different superscript letters indicate significant differences between fertilisation treatments (*p* < 0.01; Duncan’s test). * *p* < 0.05; ** *p* < 0.01; *** *p* < 0.001; ns, not significant (ANOVA), for interactions with and between nitrogen and sulphur fertilisation. Nitrogen uptake was highest in plants fertilised with N_240_S_40_ (225 ± 7 kg N ha^−1^), whereas it was significantly lower in plants grown with the same nitrogen but without sulphur (N_240_S_0_; 192 ± 7 kg N ha^−1^) (Table 2). There were no effects of sulphur fertilisation on nitrogen uptake in plants fertilised at the standard nitrogen rate of 180 kg N ha^−1^.

**Table 3:** The effects of nitrogen and sulphur fertilisation on tissue nitrogen and sulphur contents and the nitrogen:sulphur ratio in the tissue for cabbage heads and wrapped leaves.

Treatments		Tissue Nitrogen		Tissue Sulphur		Nitrogen:Sulphur Ratio	
(kg ha^−1^)		(g kg^−1^ Dry Matter)		(g kg^−1^ Dry Matter)			
Nitrogen	Sulphur	Heads	Wrapped Leaves	Heads	Wrapped Leaves	Heads	Wrapped Leaves
0	0	15.2 ± 0.6 ^c^	25.1 ± 1.1 ^c^	2.9 ± 0.1 ^d^	3.7 ± 0.4 ^c^	5.2 ± 0.4 ^c^	7.4 ± 1.1 ^a^
180	0	25.1 ± 0.6 ^b^	35.5 ± 1.2 ^b^	3.8 ± 0.2 ^c^	6.5 ± 0.8 ^b^	6.5 ± 0.3 ^b^	6.0 ± 1.0 ^ab^
	40	25.3 ± 0.7 ^b^	36.7 ± 1.3 ^ab^	4.3 ± 0.1 ^b^	9.1 ± 0.3 ^a^	5.9 ± 0.1 ^bc^	4.4 ± 0.3 ^bc^
240	0	27.1 ± 0.8 ^a^	40.0 ± 1.1 ^a^	3.6 ± 0.1 ^c^	6.4 ± 0.3 ^b^	7.6 ± 0.3 ^a^	6.0 ± 0.6 ^ab^
	40	27.8 ± 0.4 ^a^	38.9 ± 1.3 ^ab^	4.7 ± 0.1 ^a^	10.8 ± 1.3 ^a^	5.9 ± 0.2 ^bc^	3.4 ± 0.5 ^c^
**Significance**							
Nitrogen		***	***	**	**	**	*
Sulphur		ns	**	***	**	**	*
Nitrogen × Sulphur		*	**	**	*	**	**

Data are the means ± standard error (*n* = 4). Different superscript letters indicate significant differences between fertilisation treatments (*p* < 0.01; Duncan’s test). * *p* < 0.05; ** *p* < 0.01; *** *p* < 0.001; ns, not significant (ANOVA), for interactions with and between nitrogen and sulphur fertilisation.

**Table 4:** Effects of nitrogen and sulphur fertilisation on nitrate contents for cabbage heads and wrapped leaves, and the nitrogen and sulphur interactions.

Treatments		Nitrate (mg kg^−1^ Fresh Weight)	
(kg ha^−1^)		Heads	Wrapped Leaves
Nitrogen	Sulphur		
0	0	141.9 ± 24.1 ^c^	114.6 ± 84.8 ^c^
180	0	1352.1 ± 41.8 ^b^	1487.8 ± 275.1 ^b^
	40	996.2 ± 88.2 ^b^	1613.3 ± 220.0 ^b^
240	0	1816.6 ± 281.5 ^a^	3127.1 ± 394.5 ^a^
	40	1361.8 ± 184.8 ^b^	2250.4 ± 324.4 ^b^
**Significance**			
Nitrogen		***	***
Sulphur		*	*
Nitrogen × Sulphur		**	**

Data are the means ± standard error (*n* = 4). Different superscript letters indicate significant differences between fertilisation treatments (*p* < 0.01; Duncan’s test). * *p* < 0.05; ** *p* < 0.01; *** *p* < 0.001; ns, not significant (ANOVA), for interactions with and between nitrogen and sulphur fertilisation.

**Table 5:** Nitrogen balance: inputs and outputs.

Treatments		Input	Output (Nitrogen Uptake by Crop-Cabbage Heads)	Nitrogen Surplus (Input-Output)	Nitrogen Use Efficiency
(kg ha^−1^)		(kg N ha^−1^)	(kg N ha^−1^)	(kg N ha^−1^)	(%)
Nitrogen	Sulphur				
0	0	4.7	51	-	-
180	0	184.7	169	15.0	91.0
	40	184.7	181	2.2	98.0
240	0	244.7	192	52.7	78.0
	40	244.7	225	19.0	92.0

Data are the means (*n* = 4).

**Table 6:** The effects of nitrogen and sulphur fertilisation on the individual glucosinolate levels determined for cabbage heads and the nitrogen and sulphur interactions.

Treatment		Glucosinolates (µmol g^−1^ Dry Weight)												
(kg ha^−1^)		Aliphatic						Indole Brassicins				Aromatic		Overall
N	S	Sinigrin	Gluconapin	Glucoiberin	Progoitrin	Glucoibervirin	Total	Hydroxygluco-	Gluco-	Neogluco-	Total	Gluconasturtiin	Total	Total
0	0	10.8 ± 1.0	1.4 ± 0.1	6.3 ± 0.7 ^ab^	1.9 ± 0.2 ^a^	0.4 ± 0.1	20.8 ± 1.8	0.3 ± 0.14	4.1 ± 0.5 ^a^	0.5 ± 0.04	4.8 ± 0.55 ^a^	0.2 ± 0.02	0.2 ± 0.02	25.9 ± 2.3
180	0	11.6 ± 0.8	1.5 ± 0.1	5.9 ± 0.4 ^b^	1.4 ± 0.1^bc^	0.7 ± 0.1	20.9 ± 1.2	0.2 ± 0.0	2.7 ± 0.1 ^c^	0.7 ± 0.1	3.7 ± 0.3 ^b^	0.3 ± 0.03	0.3 ± 0.03	23.9 ± 1.5
	40	13.2 ± 1.3	1.5 ± 0.1	7.9 ± 0.9 ^a^	1.6 ± 0.2 ^ab^	0.7 ± 0.1	24.8 ± 2.5	0.3 ± 0.0	3.9 ± 0.3 ^ab^	0.7 ± 0.1	4.8 ± 0.6 ^a^	0.4 ± 0.1	0.4 ± 0.1	30.0 ± 3.1
240	0	11.1 ± 1.1	1.4 ± 0.1	5.4 ± 0.6 ^b^	1.3 ± 0.1 ^bc^	0.6 ± 0.1	19.8 ± 1.9	0.2 ± 0.0	3.0 ± 0.1 ^bc^	0.6 ± 0.1	3.9 ± 0.5 ^b^	0.3 ± 0.05	0.3 ± 0.05	24.0 ± 2.4
	40	10.8 ± 0.9	1.4 ±0 .04	6.4 ± 0.4 ^ab^	1.2 ± 0.1 ^c^	0.7 ± 0.1	20.5 ± 1.4	0.17 ± 0.02	2.6 ± 0.2 ^c^	0.6 ± 0.1	3.4 ± 0.3 ^c^	0.3 ± 0.0	0.3 ± 0.0	24.2 ± 1.7
**Significance**														
Nitrogen		ns	ns	ns	ns	ns	ns	Ns	**	ns	**	ns	ns	ns
Sulphur		ns	ns	*	**	ns	ns	Ns	ns	ns	ns	ns	ns	ns
N × S		ns	ns	**	***	***	ns	Ns	***	ns	ns	ns	ns	ns

Data are the means ± standard error (*n* = 4). Different superscript letters indicate significant differences between fertilisation treatments (*p* < 0.01; Duncan’s test). * *p* < 0.05; ** *p* < 0.01; *** *p* < 0.001; ns, not significant (ANOVA), for interactions with and between nitrogen and sulphur fertilisation.

**Table 7:** Chemical and physical properties of the soil of the Dolnje Brezovo experimental area used in the present study.

Parameter	Units	Value
pH (0.01 M CaCl_2_:H_2_O, 1:5)	-	7.3
Total organic carbon content	mg/kg	19,000
Total nitrogen content	mg/kg	1600
Phosphate (ammonium lactate method)	mg/kg	250
Potassium (ammonium lactate method)	mg/kg	173
Sulphur (total) (EDXRF spectrometry)	mg/kg	180
C/N weight ratio	-	11.9
**Soil fraction content**	(%)	
Sand		55.3
Silt		33.2
Clay		11.5
Soil texture	-	Sandy loam

## Data Availability

All data pertaining to this study is being held in computers owned by the University of Ljubljana, Ljubljana, Slovenia, under the control of the IP team.
